# Wielding a gun increases judgments of others as holding guns: a randomized controlled trial

**DOI:** 10.1186/s41235-020-00260-3

**Published:** 2020-11-16

**Authors:** Jessica K. Witt, Jamie E. Parnes, Nathan L. Tenhundfeld

**Affiliations:** 1 Department of Psychology, Colorado State University, Fort Collins, CO 80523 USA; 2 University of Alabama in Huntsville, Huntsville, AL USA

**Keywords:** Gun perception, Gun use, Perceptual biases, Personality, Individual differences

## Abstract

The gun embodiment effect is the consequence caused by wielding a gun on judgments of whether others are also holding a gun. This effect could be responsible for real-world instances when police officers shoot an unarmed person because of the misperception that the person had a gun. The gun embodiment effect is an instance of embodied cognition for which a person’s tool-augmented body affects their judgments. The replication crisis in psychology has raised concern about embodied cognition effects in particular, and the issue of low statistical power applies to the original research on the gun embodiment effect. Thus, the first step was to conduct a high-powered replication. We found a significant gun embodiment effect in participants’ reaction times and in their proportion of correct responses, but not in signal detection measures of bias, as had been originally reported. To help prevent the gun embodiment effect from leading to fatal encounters, it would be useful to know whether individuals with certain traits are less prone to the effect and whether certain kinds of experiences help alleviate the effect. With the new and reliable measure of the gun embodiment effect, we tested for moderation by individual differences related to prior gun experience, attitudes, personality, and factors related to emotion regulation and impulsivity. Despite the variety of these measures, there was little evidence for moderation. The results were more consistent with the idea of the gun embodiment effect being a universal, fixed effect, than being a flexible, malleable effect.

## Introduction

The false perception that another person is holding a gun leads to fatal outcomes, such as the shooting of an unarmed victim (Hall et al. 2016). This is demonstrated in many real-world examples, such as in 1999 with Amadou Diallo, who was shot 41 times by New York City police after his wallet was misperceived as a gun (Cooper 1999), and in 2018 with Stephon Clark, who was shot 8 times in his own backyard after officers misperceived his cell phone as a gun (Robles and Del Real 2018). The false perception even extends to the young, such as Tamir Rice, 12 years old, who was playing with a pellet gun when he was shot by two police officers who perceived the toy gun to be real (McCarthy 2014). What leads to these fatal false perceptions? One contributing factor could be the influence of stereotypes on decisions to shoot, such as the race of the subject one is making a decision about (Correll et al. 2016; Kahn and Davies 2017). People are more likely to misperceive a Black person or a Muslim person as holding a gun than a White person (Essien et al. 2017; Hall et al. 2016; Kahn and Davies 2017). However, it is unlikely that the influence of stereotypes completely explains these fatal false perceptions.

Another factor informing the decision to shoot is whether the person making the decision to shoot is wielding a gun (Witt and Brockmole 2012). More specifically, when person A is wielding a gun, they are more likely to indicate that person B is wielding a gun than when person A is holding a neutral object, such as a ball. This effect holds regardless of whether person B is holding a gun or a neutral object. The bias to judge another person as holding a gun when wielding a gun oneself is referred to as the *gun embodiment effect*.

Research supporting that wielding a gun influences the perception of others provides support for the embodied social cognition theory (Smith and Semin 2004, 2007). According to this theory, social cognition is influenced by embodied factors such as body structure, posture, movements, and potential for action. While there is growing support for this research (for review see Fincher-Kiefer 2019; Shapiro 2019), there is also growing concern whether the effect can be replicated (e.g., Wagenmakers et al. 2016). The purpose of the current research was to conduct a high-powered study of the gun embodiment effect to attempt to replicate the effect, determine its stability, and precisely estimate its effect size. In addition, we conducted a preliminary investigation into whether the gun embodiment effect is modulated with respect to individual differences, including trait-based differences such as personality or experience-based differences such as video game experience.

### Guns, social cognition, and embodiment

Prior research on guns has shown effects on both increasing aggressive behavior and biases in the perception of the guns. For instance, the mere placement of a gun in a room leads to more aggressive behavior (Berkowitz and LePage 1967). Guns, like other violent-related stimuli such as violent television (Eron et al. 1972), violent media clips (Anderson et al. 2010; Berkowitz 1984), and violent video games (Anderson and Bushman 2001; Calvert et al. 2017), lead to more aggressive behaviors because they prime aggressive scripts or schemas (Anderson et al. 1998; Berkowitz 1990). In other contexts, rather than being a subtle manipulation of placement, the perception of guns has been directly examined. The accuracy with which guns are perceived as being held by others is influenced by the race of the depicted person: observers are more biased to perceive guns—even when guns are not present—in the hands of Black individuals compared with White individuals (Correll et al. 2002; Eberhardt et al. 2004; Greenwald et al. 2003; Payne 2001).

Previously, we found that when holding a gun, participants were more biased to perceive others as holding guns compared with when they held a neutral object, such as a rubber ball (Witt and Brockmole 2012). This effect was independent of the race of the depicted person, suggesting a new gun-related social perception bias based on the action capabilities of the observer. In addition, the mere placement of a gun in the room did not lead to a similar increased bias to see guns. This suggested that this effect did not share the same mechanisms as the ones that lead to more aggressive behaviors based solely on the presence of a gun, but that the effect was instead specific to the wielding of the object.

The manipulation of changing participants’ bodies and/or action capabilities by arming them is situated in embodied social cognition theory (Meier et al. 2012; Niedenthal et al. 2005; Smith and Semin 2004, 2007). According to this approach, the body and its capabilities for action influence social cognitive processes such as perception of others’ emotions, intentions, facial expressions, bodily postures, gestures, and actions. For example, participants’ facial postures were manipulated by having them hold a pen in their lips (which stimulates muscles for frowning) or in their teeth (which stimulates muscles for smiling). Facial posture influenced ratings of the emotional expressions of others as being sadder versus happier, respectively (Blaesi and Wilson 2010; Niedenthal et al. 2000). In addition, pen-based manipulations of facial posture biased ratings of the funniness of cartoons: cartoons were perceived to be funnier when mimicking a smile compared to a frown (Strack et al. 1988, but see Wagenmakers et al. 2016). Facial posture also affected recall of affective material: those induced to a smiling-like posture recalled more positive than negative material (Laird et al. 1982).

Head movements also influence social cognition: participants who engaged in vertical head bopping (akin to nodding) were more likely to agree with the content of radio broadcasts than those who engaged in horizontal head movements (akin to shaking one’s head; Wells and Petty 1980). Body posture similarly induces effects in social cognition: slumping reduces feelings of pride over one’s achievements (Stepper and Strack 1993). Body posture has also been manipulated to induce approach versus avoidant behaviors. Pushing (such as pressing down on a table), which simulates an avoidant behavior, leads to more negative ratings of stimuli (Cacioppo et al. 1993), and faster classification and recognition of negative words than pulling, which simulates an approach behavior (Chen and Bargh 1999; Markman and Brendl 2005; Neumann and Strack 2000; Wentura et al. 2000).

Other bodily states also produce effects on social cognition. Body temperature influences perceived social proximity: holding a warm drink or being in a warm room leads to the perception of the distance to others as being closer compared with holding a cold drink or being in a cold room (Ijzerman and Semin 2010). Bodily cleanliness (e.g., washing one’s hands) attenuates feelings of guilt (Zhong and Liljenquist 2006) and eases judgments of the misdeeds of others (Schnall et al. 2008).

In addition to manipulations of bodily states, individual differences related to the body also influence social cognitive processes. In particular, individual differences related to motor expertise influence the perception of others. The brain activity in ballet experts was increased when watching ballet dancers compared with Capoeira dancers (Calvo-Merino et al. 2005). For experts in Capoeira, the reverse pattern was found. Similarly, experts in hockey produced differential brain activity and heightened understanding when reading sentences describing hockey movements (Beilock et al. 2008).

### Replication crisis

In recent years, there is growing concern about the replicability of psychology experiments in general (Open Science Collaboration 2015), and experiments supporting embodied social cognition theory in particular (Bohannon 2014). There have been many failures to replicate embodied social cognition findings including the effect of washing hands on morality judgments (Johnson et al. 2014), the effect of holding a pen in one’s mouth on perceived humor in cartoons (Wagenmakers et al. 2016), and the effect of holding a hot therapeutic pack on prosocial behavior (Lynott et al. 2014).

Concerns about replication are particularly relevant for the gun embodiment effect because the original studies were underpowered (Witt and Brockmole 2012). The effect size in their Experiment 2 was *d*_z_ = 0.366. To achieve 80% power to detect an effect of this size, the required sample size is *N* = 61 participants. The experiment had only 38 participants, which achieves a power of only 59%. In addition, the *p* values for the gun embodiment effect obtained in the previous paper were 0.02, 0.03, 0.10, and 0.01. These values do not inspire much confidence in the gun embodiment effect. A *p*-curve analysis contends that effects that are likely to be true should result in a right-skewed *p*-curve, meaning that most *p* values should be closer to 0.01 than to 0.04 (Simonsohn et al. 2014). Currently, the data supporting the gun embodiment effect do not meet the *p*-curve criterion. One goal of the current experiment was to run a high-powered study of the gun embodiment effect with an emphasis on estimating the size of the effect. Larger sample sizes are needed to more accurately estimate effect size.

### Individual differences

In addition to trying to estimate the effect size of the gun embodiment effect, we also conducted a preliminary investigation into whether any individual differences moderate the gun embodiment effect. Understanding whether these embodiment effects can be moderated is critical because, if moderators exist that influence the extent to which embodiment influences social cognition, this is evidence that the pathway from embodiment to social cognition is flexible and malleable. This would provide leverage points for interventions to reduce false gun perceptions that lead to fatal shootings. In contrast, if the effect is consistent across all individual differences, this suggests this embodiment effect on social cognition is automatic and stable. Such a universal, fixed bias might be difficult to alleviate with training.

There are instances in the literature showing modulation of embodiment effects (Landau et al. 2010; Meier et al. 2004, 2012; Sherman and Clore 2009; Sugovic and Witt 2013). These studies lend credence to the idea that embodied pathways for social cognition are affected by the context of the individual and the situation. Yet, theories of embodiment continue to explore effects as though the pathways were universal, stable, automatic, and fixed. One reason may be that early research focused on demonstrating the importance of embodied-related factors. However, the time has come to move beyond demonstrations and explore the underlying mechanisms and potential moderators of embodiment effects in greater detail. This is especially relevant given that many of the prior findings have been challenged with respect to their replicability (Wagenmakers et al. 2016). Understanding the underlying mechanism and potential for moderation of the gun embodiment effect would help to determine when embodiment effects should and should not be present.

As an initial investigation, we selected a wide range of individual differences measures including stable traits such as the Big 5 personality traits and experience-dependent measures such as being a gun owner or playing first-person shooter video games. The purpose of the study is to determine whether *any* moderators exist, which speaks to the nature of the gun embodiment effect, rather than to test specific hypotheses about particular relationships. Nevertheless, certain measures were motivated by theoretical considerations. One such measure was prior experience, either with guns themselves or with first-person shooter video games. Motor experience plays a central role in embodiment literature, with people who have expertise showing stronger effects of embodiment when watching others (Calvo-Merino et al. 2005) and when comprehending action-based sentences (Beilock et al. 2008). Thus, we expected that people with prior gun or gaming experience would show a stronger gun embodiment effect.

We also predict that the certain personality traits such as neuroticism and extraversion would moderate the gun-embodiment effect. Individuals who score higher on neuroticism are more likely than those who score low to experience anxiety, anger, and depressed mood especially when under stress (Griffith et al. 2010), and are more likely to interpret ambiguous situations as threatening (Lommen et al. 2010). The specific hypothesis in this study is that participants who score high on neuroticism will exhibit a stronger gun-embodiment effect because the gun will likely induce a more stressful situation that will promote more negative emotions, which may lead to an increased bias to see guns. Individuals that score high on measures of extraversion are warm, gregariousness, assertive, hyperactive, excitement seeking, and tend to experience positive emotion when compared to individuals that score low on extroversion (Jylhä and Isometsä 2006; Lucas et al. 2000). Those high in extraversion are also more likely to struggle inhibiting prepotent responses (Helmers et al. 1997). As such, we hypothesized that participants who score higher on extraversion will exhibit higher gun-embodiment effects.

We also included a behavioral measure of impulsivity. Impulsivity involves a tendency to act with little to no forethought, reflection, or consideration of consequences (Evenden 1999). High behavioral impulsivity is characterized by behavior that is poorly conceived, hastily expressed, unnecessarily risky or inappropriate to context or situational factors, and often results in undesirable consequence. We predicted that participants who are higher in impulsivity would exhibit higher gun-embodiment effects. This would be consistent with the idea that merely wielding a gun creates a prepotent response to shoot, which would be more difficult for participants with high impulsivity to inhibit.

We explored whether participants who have more positive attitudes toward guns will show stronger gun-embodiment effects. If so, this raises the possibility that positive attitudes modulate embodiment such that people who have more positive attitudes are more likely to incorporate the gun into their bodies and thus perceive others accordingly. Such a finding would go strongly against the perhaps implicit ideas of embodiment being automatic and instead suggest a component that can be intentional.

Just as attitudes about guns might moderate the gun-embodiment effect, attitudes toward one’s ability to exert control over the environment might also moderate the effect. Participants with greater internal locus of control could exhibit higher gun-embodiment effects because these individuals feel that they have more control over the environment and thus may be more influenced by changes in their action capabilities and how those changes impact what they can. This result would demonstrate that attitudes toward one’s ability to act in and exert control over the environment are relevant for embodied effects.

### Current study

The study reported on herein had two aims. The primary aim was to replicate the gun embodiment effect (the effect of wielding a gun on biases to report seeing a gun found in previous research; Witt and Brockmole 2012) using a larger sample of the population. A secondary aim was to identify whether individual differences, including differences in the Big Five personality traits, sensation seeking, impulsivity, emotion dysregulation, locus of control, and experience with, as well as attitudes regarding, guns moderate the previously established gun embodiment effect.

## Experiment 1

### Method

#### Participants

Participants were 212 students at Colorado State University (CSU) who received course credit in exchange for their participation. This sample size gives us 80% power to find statistical significance at *α* = 0.05 for a one-sample *t*-test with an effect size of *d* = 0.19 and also 80% power to find statistical significance for a correlation of *r* = 0.19. The sample was 71.1% female, 84.3% White, 82.9% non-Hispanic, and had a mean age of 19.2 years (SD = 1.54). The study had the approval of the Colorado State University Institutional Review Board.

#### Procedure

Participants completed the gun perception task and a variety of individual differences measures.

#### Gun perception task

**Fig. 1:**
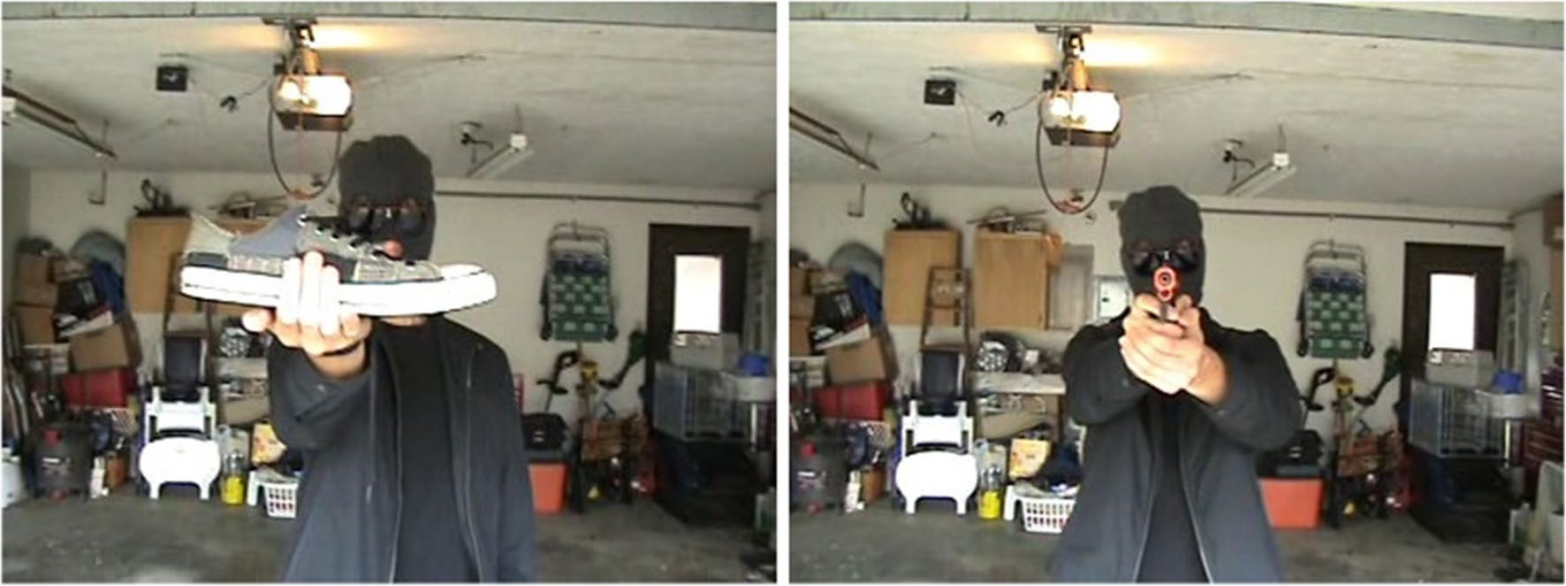
Sample stimuli of the shoe (left) and gun (right) conditions

The first task participants completed was the gun perception task. For this task, participants stood approximately 5 feet away from a 55″ liquid crystal display television screen and responded to a series of images. The images contained a White person, dressed in black and wearing a black ski mask, who was holding either a black gun pointed at the camera or a white shoe held perpendicular to the line of site to the camera (see Fig. 1). The images were taken from screen shots of movies filmed in 19 different locations. For each location, one movie was made with the gun and another with the shoe, and the screen shots selected attempted to make as much of the scene and man’s stance identical between the two images. The 38 images were the same as those used in the previous study (Witt and Brockmole 2012).

**Fig. 2:**
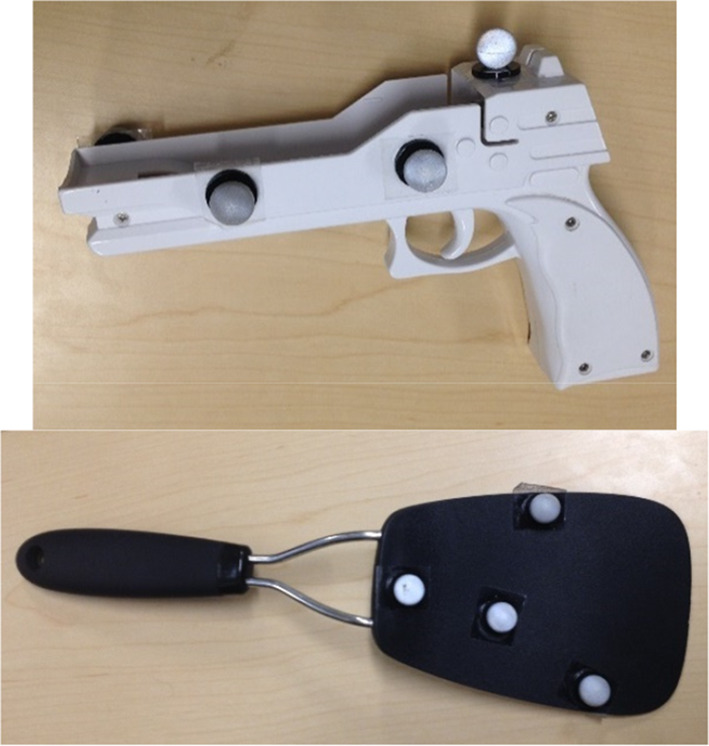
Objects held during the experiment. The markers were affixed to the objects to track the participants’ movements

Participants completed two blocks of trials, which varied only in the object they used to respond. For one block, participants held a Wii gun (21.6 cm length, 3.8 cm width, 12.7 cm height). For the other block, participants held a spatula (34.3 cm length, 10 cm width, 3.1 cm height, Oxo brand). Motion tracking markers were affixed to each object to track the trajectories of the participants’ movements (see Fig. 2). Movements were measured using a Vicon 3D motion tracking camera system (Vicon Nexis 2.0, Vicon Bodybuilder 3.6.1, and Bonita 10 Camera) that tracked the location of the markers on each object in *X*, *Y*, and *Z* coordinates. The *Z*-coordinate corresponds to up and down and was the coordinate used to determine movements (see “Data Preprocessing” section). The first object held was counterbalanced across participants.

**Fig. 3:**
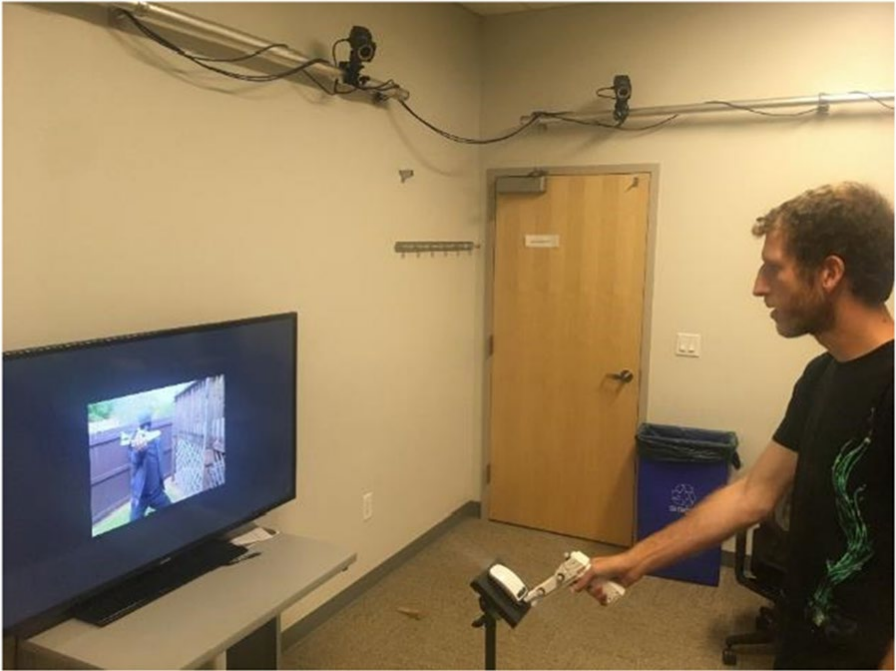
Photograph of the experimental set up for the gun task

At the start of each trial, the participant placed the held object (gun or spatula) onto a wireless mouse that was affixed to a music stand (see Fig. 3). Depressing the mouse button was necessary to start the trial. An initial series of practice trials used arrows to help participants become familiar with the task. Upon pressing the mouse button, an up or down arrow would appear, and participants would move their object off the mouse and point up or down as indicated by the arrow. They were instructed to initiate their response only once they knew which direction to move and to make the movement in one smooth motion. Participants were reminded of this instruction throughout the experiment as needed. Participants completed 12 practice trials, 6 in each direction, and order was randomized.

For the test trials, text on the screen said “Get ready” to instruct participants to place the object onto the mouse. Once the mouse was depressed, a blank screen with a fixation cross was presented for a randomly selected delay between 500 and 1000 ms. The delay duration was randomized to minimize anticipatory responses. One of the test images was then presented. As displayed on the screen, it measured 57.5 × 96.8 cm. The image remained on the screen for 850 ms or until participants released the mouse button. Participants moved up when they saw a gun and moved down when they saw a shoe. Then a blank screen with three asterisks was presented until the research assistant coded the movement. The research assistant coded movements as 1 for up, 2 for down, and 3 for an error such as slipping off the mouse or reversing directions, then the next trial began. Due to a programming error, the experimenter’s entry was not recorded for the first group of participants. For analysis, we used the motion tracking data unless it was missing due to the cameras being unable to detect the sensors, in which case we used the experimenter’s entry instead.

Participants completed two blocks of trials, one for each held object. Order of held object was counterbalanced. Each block contained 152 trials, which included two presentations of each of the 38 images and of a mirror-reversal version of each. Order within block was randomized.

#### Measures of individual differences

Participants then completed several self-report measures of individual factors via laptop computer and the Qualtrics survey platform (*Qualtrics *Inc 2019). First, participants indicated how often they use a gun. Possible responses were ‘never’, ‘less than once a year’, ‘1 to 5 times per year’, ‘6 to 12 times per year’, and ‘13 or more times per year’. The frequency was later dichotomized into ‘never’ and ‘at least once’ for purpose of the analysis. Participants were also asked whether they play gun-oriented video games 4 or more hours per week, and responded with either a yes or no. Following, participants completed the Gun Attitudes Scale (Tenhundfeld et al. 2020), which measured a general attitude toward gun use. Next, they completed an assessment of the Big Five Personality Traits (John and Srivastava 1999), which measured extroversion, conscientiousness, agreeableness, neuroticism, and openness to experience. The Sensation Seeking Personality Type (SSPT) scale (Conner 2019) was used to assess sensation seeking. The SSPT contains two subscales, experience seeking, which measures an individual’s propensity for seeking novel experiences, and risk seeking, which measures an individual's willingness to take risks. The UPPS + P scale (Lynam et al. 2006; Whiteside and Lynam 2001) was administered to measure impulsivity-related traits including positive urgency, negative urgency, lack of perseverance, and lack of premeditation. Emotion dysregulation was measured using the Difficulty with Emotion Regulation Scale (DERS) (Gratz and Roemer 2004), which assesses various aspects of emotion regulation, including emotional clarity, emotional awareness, emotionally driven impulsivity, emotional non-acceptance, difficulty engaging in goal-directed behavior while emotionally distressed, and access to emotion regulation strategies. Finally, participants completed the Levenson Multidimensional Locus of Control Scales (Levenson 1973), which measured a participant’s locus of control related to Internal Locus of Control, Powerful Others, and Chance.

Following the surveys, participants completed the Stop Signal Task, a behavioral measure known to correlate with inhibition and impulsive responding (Lappin and Eriksen 1966; Li et al. 2006). During this task, participants were shown a large, gray box on the computer monitor. In the box, a target moved from one side of the screen to the other. Participants were instructed to click on the target before it reached the other side. On 20% of the trials a whistle sounded at varying delays (50, 150, 250 and 350 ms) after trial initiation. On such trials the participant was instructed to inhibit the click response. A total of 120 trials (24 stop signal, 96 no signal) were included in each session. The percent correct responses were calculated for trials without the stop signal, and for each stop signal delay. Percent correct is inversely related to impulsivity.

### Data preprocessing and planned analyses

For each participant, there were 3 data files: two motion tracking files that coded the x, y, and z-coordinates for each object held (spatula and gun) and one file that coded the stimuli, response time (RT) and experimenter’s coding of the movement. We used custom code to align the three data files. Alignment could not be achieved for 19 participants due to noise or issues with the motion tracking data. For 9 of these participants, hand-coded responses were available and used; data were excluded for the remaining 10 participants. Motion tracking data were missing for one or both conditions for 20 participants, so their data were excluded. Of the 182 participants in the final analysis, trials with no movement were excluded. This comprised less than 0.5% of the data.

The next step was to classify the movement on each trial. Movements were classified based on the vertical position of the object during each trial. Thresholds for moving up and for moving down were calculated as 30% above and below the median location. Trials with movements that did not reach either threshold were categorized as no movement trials and excluded. Trials with movements that reached both thresholds were categorized as reversals and coded as errors. Experimenter entry was used to verify coded motion tracking data and resolve any discrepancies.

Data analyses were done in R (R Core Team 2017). A critical question was whether there was a difference in responses when holding a gun versus holding a spatula. Scores were computed for each of the hold conditions (gun and spatula) and analyzed with a paired-samples *t*-test. Another critical question was whether the gun embodiment effect correlated with other individual differences and these data were analyzed with Pearson correlations. For both the *t*-tests and the correlations, we calculated Bayes factors (BFs) using the R BayesFactor package with the default Cauchey prior (Morey et al. 2014). BFs are presented as the Bayes factor in favor of the alternative (BF_10_), so values greater than 1 are evidence in favor of the alternative over the null hypothesis with values greater than 3 and greater than 10 constituting substantial and strong evidence, respectively (Jefferys 1990; Lakens 2016). Values less than 1 are evidence in favor of the null over the alternative hypothesis, and values less than 0.33 and less than 0.10 are considered substantial and strong evidence for the null hypothesis over the alternative, respectively. Cohen’s *d*_*z*_ was calculated using the cohensD function in the lsr package (Navarro 2015).

#### Multiverse analysis plan

To eliminate effects of experimenter degrees of freedom, we conducted a multiverse analysis (Steegen et al. 2016). For a multiverse analysis, the same test (e.g., the paired-sample *t*-test) is calculated many times for each possible variation such as variations in the dependent measures used or variations in outlier exclusion. For the current study, there were four forks in the “garden of forking paths” (Gelman and Loken 2013). One fork concerned the dependent measure, which could be accuracy, signal detection measures of *A*′ and *B*″, or reaction time. The other 3 forks concerned outlier exclusion. For reaction times, outliers can be determined on the basis of raw reaction times, mean reaction times, or differences in reaction times. For other measures, outliers can be determined on the basis of mean scores or differences in scores. At each fork, we conducted a multiverse with three criteria: no outliers excluded, outliers beyond 3 times the interquartile range (IQR), and outliers beyond 1.5 times the IQR. The multiverse analysis showed consistent patterns regardless of the outlier criteria selected (Additional file 1). This suggests the effects (or lack thereof) are robust to outlier exclusion. Thus, for ease of presentation, only one path for each dependent measure is presented in the Results section. This path used the criteria of excluding RTs that were beyond 1.5 times the subject- and stimulus-specific IQR and excluding both mean scores and difference scores that were beyond 1.5 times the group’s IQR for each dependent measure assessed. Because this criterion was applied based on the specific measure being analyzed, different participants were excluded, and the degrees of freedom differed across the various analyses.

### Results

The analyses are split into two sections that coincide with the two aims: replicating the gun embodiment effect and determining whether individual differences moderate the effect.

#### Gun embodiment effect

**Fig. 4:**
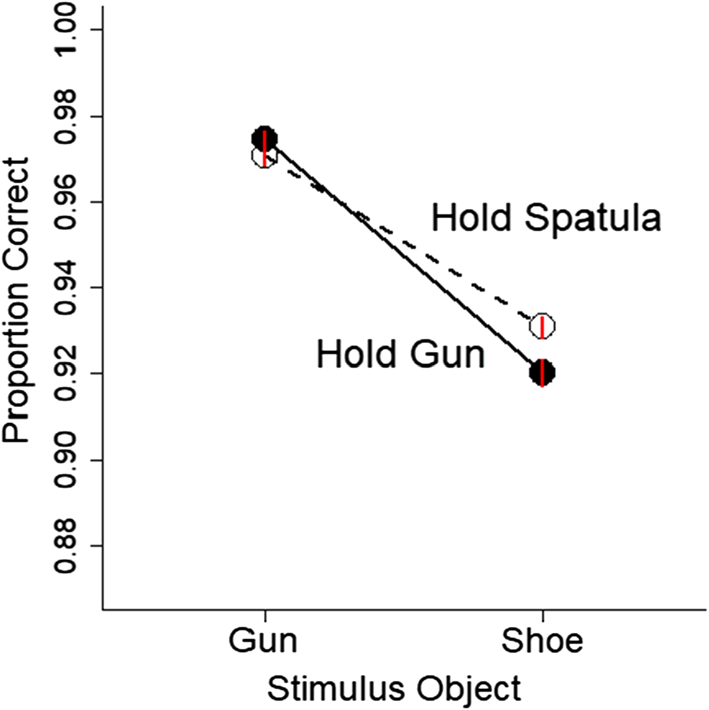
Proportion correct is plotted as a function of the hold condition and the stimulus object (gun and shoe). Error bars are 1 SEM calculated within-subjects and are approximately the same size as the symbols

The gun embodiment effect was originally defined as the bias to report the presence of a gun more often when holding a gun than when holding a neutral object. To quantify this bias, we calculated the nonparametric signal detection theory measures of *A*′ and *B*″. *A*′ provides a nonparametric measure of discriminability, which refers to the ability to distinguish when a gun versus a shoe is present. *B*″ provides a nonparametric measure of bias, which refers to the tendency to report that a gun (or a shoe) is present. For each participant for each hold condition, we calculated hit rates and false alarm rates based on the proportion correct scores (see Fig. 4). Hits refer to when a gun was present and the participant reported that a gun was shown in the picture (i.e., an up movement). False alarms refer to when a shoe was present but the participant incorrectly reported that a gun was shown. From the hit and false alarm rates, we calculated *A*′ and *B*″ scores based on formulas in Stanislaw and Todorov (1999). *B*″ scores could not be computed for 4 participants due to perfect performance (100% hits and 0% false alarms) in one of the conditions.

**Fig. 5:**
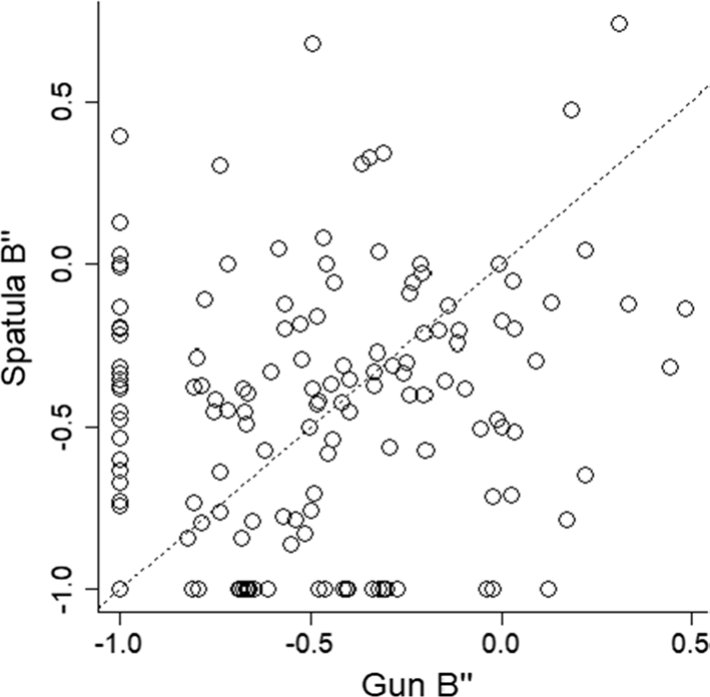
*B*″ scores when holding the spatula are plotted as a function of *B*″ scores when holding the gun. Each point corresponds to one participant. Lower *B*″ scores indicate more bias to respond that a gun is present. The line represents equality between the two conditions. The gun embodiment effect would be expressed as more points above the equality line, which would mean more bias to respond that a gun is present in gun condition than the spatula condition

We conducted a paired-samples *t*-test between the *B*″ scores when holding the gun and the B’’ scores when holding the spatula. The effect was not statistically significant, and the Bayes factor showed 7 times more support for the null hypothesis than the alternative, *t*(146) = 0.95, *p* = 0.34, BF = 0.14, *d*_*z*_ = 0.08, 95% CI [− 0.08, 0.24]. The original paper (Witt and Brockmole 2012) reported a significant difference in *B*″ scores between holding a gun versus a neutral object. The current analysis showed a clear failure to replicate this effect (see Fig. 5).

**Fig. 6:**
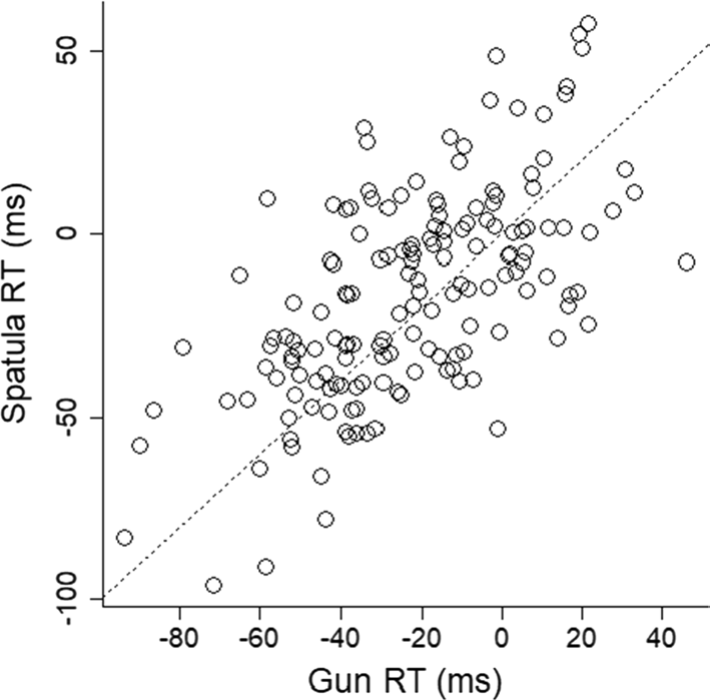
RT difference scores when holding the spatula are plotted as a function of RT difference scores when holding the gun. Larger difference scores indicate increased time to respond when seeing a shoe relative to responding when seeing a gun. The line represents equality between the two conditions. The gun embodiment effect would be expressed as more points below the equality line, which would mean slower responses to seeing a shoe (vs a gun) in the hold gun condition compared with the hold spatula condition. These data were calculated using the 1.5xIQR criteria for outlier decisions (second to left column and bottom row in the multiverse)

**Fig. 7:**
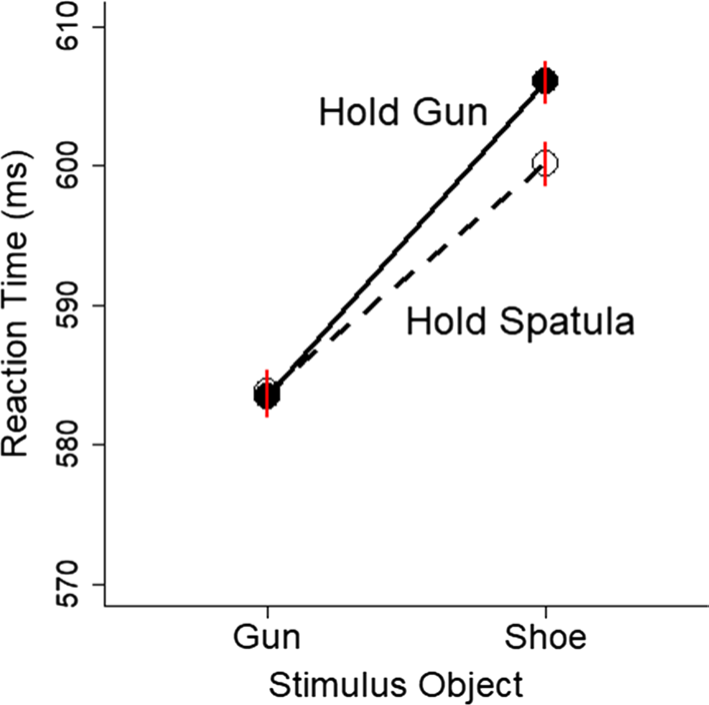
Mean reaction time is plotted as a function of the stimulus object (gun and shoe) and the hold condition (gun and spatula). Error bars are 1 SEM calculated within-subjects. The gun embodiment effect is shown by the steeper solid line compared with the dashed line. This shows slower reaction time to respond that a shoe is present versus gun is present when holding a gun compared with when holding a spatula

The original research showed a gun embodiment effect on the bias scores but no effect on reaction times. In contrast, the current study showed no effect on the bias scores but an effect on reaction times (RTs). We calculated mean RTs for each participant for each hold condition and for each stimulus condition, and then calculated the difference in mean RTs to respond to a gun compared with a shoe for each hold condition. These two difference scores were analyzed with a paired-samples *t*-test to determine whether the speed to respond to a gun versus a shoe differed when holding a gun versus a spatula. The effect was statistically significant, and the Bayes factor showed over 10 times more support for the alternative than the null hypothesis, *t*(159) = 3.29, *p* = 0.001, BF = 14.98, *d*_z_ = 0.26, 95% CI [0.10, 0.42], *M*_diff_ = 6 ms, 95% CI [2, 10 ms], 59% of participants showed a positive effect (see Figs. 6, 7).

The data also showed a significant gun embodiment effect on accuracy (see Fig. 4). Accuracy was calculated as the difference score in the proportion of correct responses when seeing a gun minus when seeing a shoe for both the hold gun and hold spatula conditions. This difference score was larger when holding a gun than when holding a spatula, *t*(142) = 3.07, *p* = 0.003, BF = 8.19, *d*_z_ = 0.26, 95% CI [0.09, 0.42], *SD*_diff_ = 1.4%, 95% CI [0.5, 2.3%], 58% of participants showed a positive effect.

To summarize the results thus far, the gun embodiment effect was present. However, the gun-embodiment effect did not express itself in bias scores, as had been found in the original research (Witt and Brockmole 2012). Instead, the gun embodiment effect expressed itself in reaction time and in accuracy. Regardless of how the gun embodiment effect was measured, the effect was small (*d*_*z*_ = 0.26 for both two measures). To achieve 80% power to obtain a *p* value < 0.05 for an effect of *d*_*z*_ = 0.26, an experiment would need approximately 118 participants (or 93 participants with a one-sided *t*-test). That power was achieved in the current study, so we next assessed whether any individual differences moderated the gun embodiment effect.

#### Individual differences as moderators of the gun embodiment effect

The next research question pertained to the universal nature of the gun embodiment effect. If no individual differences measures moderate the magnitude of the gun embodiment effect, this would be evidence for a universal and fixed effect. In contrast, if some individual differences cause the gun embodiment effect to amplify, or to be eliminated, this would suggest the effect is malleable and flexible.

**Fig. 8:**
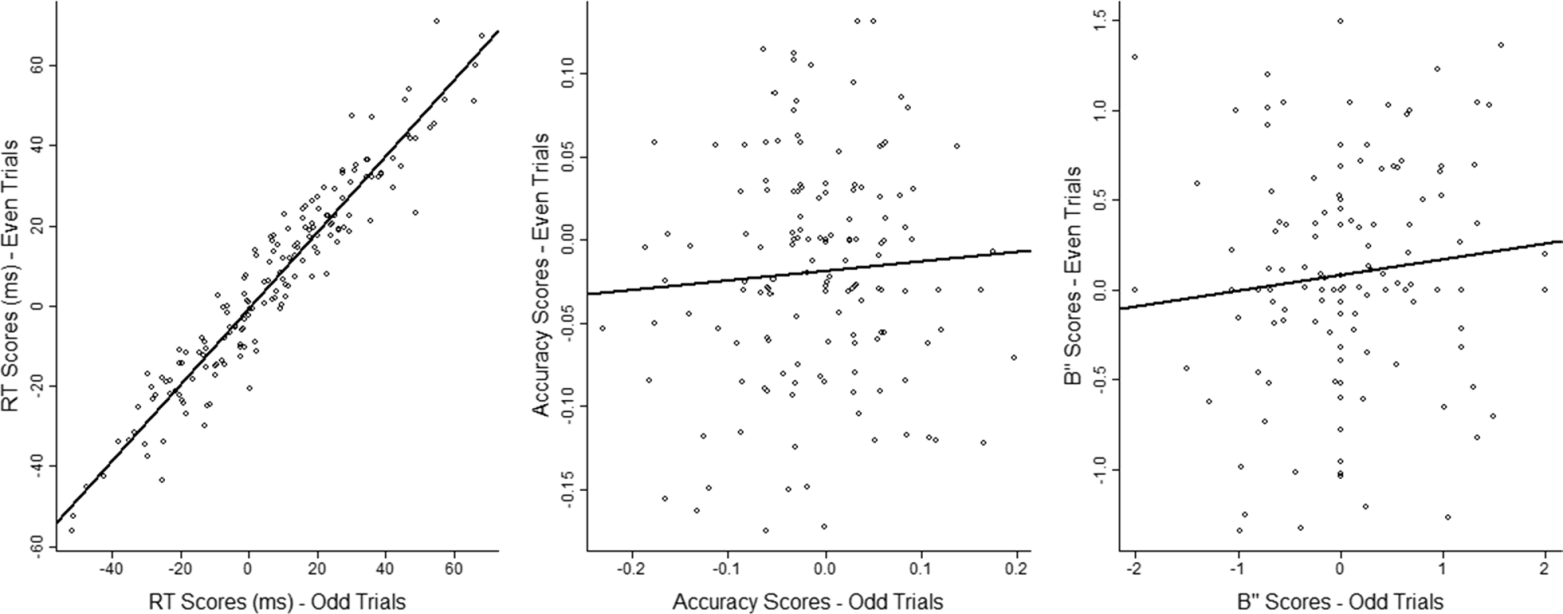
Correlations between odd and even trials for three measures of the gun embodiment effect: reaction time (RT), accuracy, and *B*″. In each panel, each symbol corresponds to the data from one individual. Each score was calculated as a difference of difference scores, so positive values indicate the gun embodiment effect

Before exploring moderators, we had to select which dependent measure to use to quantify the gun embodiment effect. To do individual differences research, it is necessary that a measure have high reliability. Also, even if a measure does not show a main effect, it can still show a correlation (Miller and Schwarz 2018). We evaluated the reliability of the gun embodiment effect as measured with reaction times, accuracy, and bias scores. For each score, we calculated the relevant difference score on the odd trials and on the even trials (split based on condition) and calculated the correlation between the two. Only RTs showed high reliability (see Fig. 8). Thus, all subsequent analysis involved only the gun embodiment effect as measured with RTs.

**Fig. 9:**
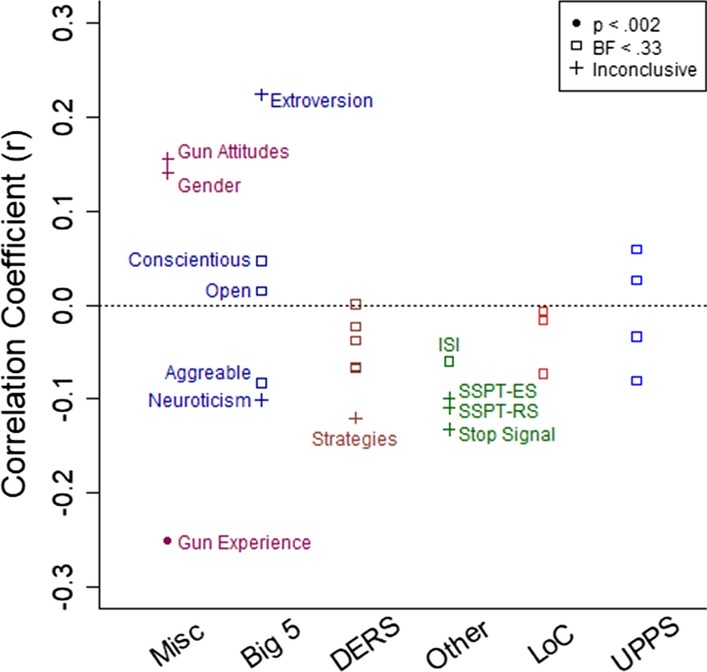
The correlation coefficient for each comparison with the gun embodiment effect measured with reaction time. The coefficients are plotted as a function of category of comparison and whether the *p* value met the criteria for statistical significance after Bonferroni correction (*p* < .002, filled circles), substantial evidence for the null over the alternative hypothesis (BF < .33, open squares), or inconclusive (crosses)

We calculated the correlation between the gun embodiment effect (measured with RT difference scores) and each of the individual differences measures. Overall, there were few correlations of any notable magnitude (see Fig. 9).

**Fig. 10:**
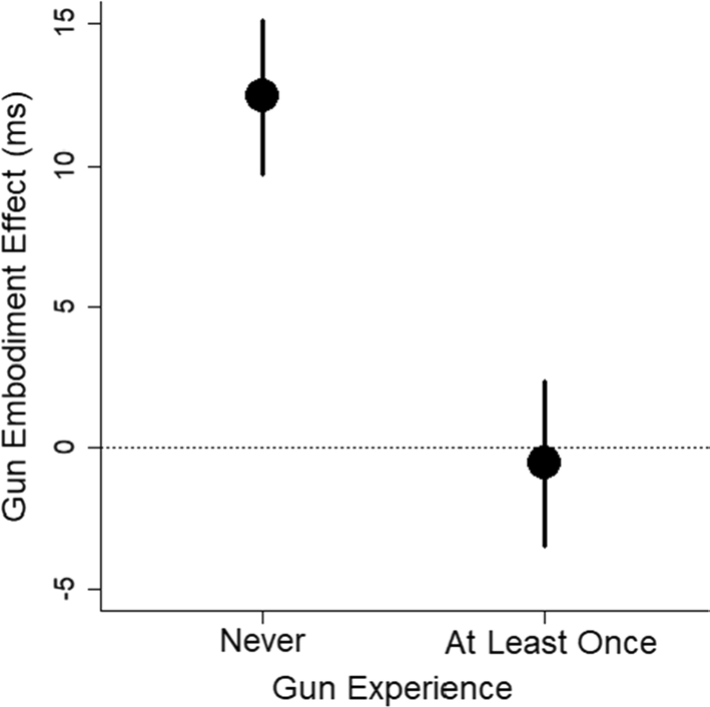
Mean gun embodiment effect (measured with reaction time) is plotted as a function of prior gun experience. Error bars are 1 SEM

The largest effect was the correlation between prior gun experience and the gun embodiment effect, *r* = − 0.25, *p* = 0.002, BF = 18. Given that gun experience was coded into binary categories of *never* and *at least once*, we ran a *t*-test. A two-sample *t*-test revealed that gun experience affected the gun embodiment effect, *t* = 3.26, df = 133.62, *p* = 0.001, BF = 15, *d* = 0.53, 95% CI [0.19, 0.87]. As shown in Fig. 10, the gun embodiment effect was present for people who had never used a gun, *t* = 4.57, *df* = 91, *p* < 0.001, BF > 1000, *d* = 0.48, 95% CI [0.26, 0.69], but the gun embodiment effect was not present for people who had used a gun, *t* = − 0.19, df = 560 *p* = 0.84, BF = 0.15, *d* = 0.03, 95% CI [− 0.23, 0.28].

**Fig. 11:**
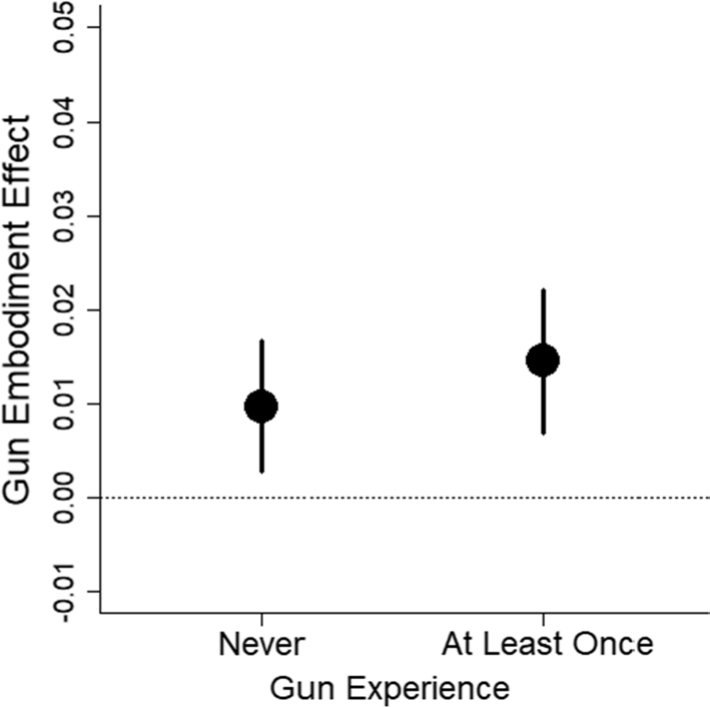
Mean gun embodiment effect (measured with proportion of correct responses) is plotted as a function of prior gun experience. Error bars are 1 SEM

That gun experience modulates the gun embodiment effect is direct evidence against the idea that the gun embodiment effect is universal and fixed. Instead, the result suggests the effect is malleable and can be modified with prior experience. However, this conclusion is not fully supported by the data. When we looked at the gun embodiment effect as measured with accuracy scores, people with prior gun experience showed some tendency toward the gun embodiment effect, *t* = 1.92, *df* = 56, *p* = 0.060, BF = 0.80, *d* = 0.25, 95% CI [− 0.01, 0.51] (see Fig. 11). This hint of an effect with accuracy scores casts doubt on whether people with prior gun experience are truly immune to the gun embodiment effect. We return to this issue in Experiment 2.

**Fig. 12:**
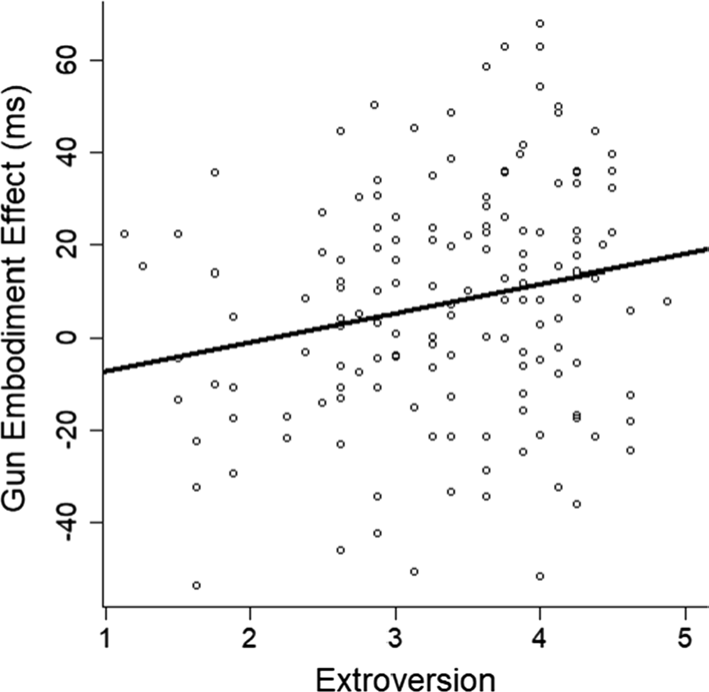
Mean gun embodiment effect (measured with reaction time) is plotted as a function of extroversion. Each circle represents one participant. Line represents linear regression

Regarding the other correlations, only one other correlation was close to significance. This was the correlation between extroversion and the gun embodiment effect, as measured with reaction time, *r* = 0.22, *p* = 0.006, *df* = 146, BF = 7 (see Fig. 12). Bonferroni correction for multiple comparisons would set the alpha equal to 0.002 (0.05/25 comparisons), in which case, this result would not be deemed significant. Bonferroni is perhaps overly conservative, although the same conclusion of failing to achieve significance resulted from Benjamini–Hochberg correction as well. However, the Bayes factor showed the data provided substantial support for the alternative over the null hypothesis. Together, the conclusions are mixed. We think it best to treat this finding as preliminary and a possible avenue for future exploration but not definitive evidence for modulation of the gun embodiment effect.

None of the other factors showed significant correlations with the gun embodiment effect. In a few cases, the correlation could be present, but we did not have enough power to detect it. We had approximately 80% power to detect a correlation of *r* = 0.23, so perhaps some of the correlations were smaller than this value (see Fig. 9).

In other cases, there was substantial evidence from Bayes factors that no correlation existed between the measure and the gun embodiment score. Measures with Bayes factors less than 0.33 (and thus have substantial evidence for the null hypothesis over the alternative hypothesis) included 5 of the 6 measures of Difficulty with Emotion Regulation scale (DERS; (Gratz and Roemer 2004), 3 of the Big 5 personality measures (agreeableness, openness, and conscientiousness), the 3 locus of control measures (Levenson 1973), and the 4 measures of impulse-related traits (Lynam et al. 2006; Whiteside and Lynam 2001). These items were some of our best guesses as to factors that would moderate the gun embodiment effect. That so few correlations emerged, and that so many even had substantial evidence for the null hypothesis, speaks to a robustness and inflexibility of the gun embodiment effect, even if the effect is small.

### Discussion

The goal of Experiment 1 was first to attempt to replicate the gun embodiment effect and second to determine whether any individual differences modulate the effect. The data showed strong support for the gun embodiment effect. However, in contrast to the previously published research (Witt and Brockmole 2012), the current results show the effect is small (*d*_*z*_ = 0.26) and the gun embodiment effect is more likely to be expressed in reaction time or accuracy rather than the nonparametric signal detection measure of bias.

Regarding the second goal, we conducted 25 various tests for modulation. Two of these tests suggested modulation. One was the personality measure of extroversion. We had predicted that people higher in extroversion would show a stronger gun embodiment effect because they are more likely to make rash decisions and to act without forethought. However, the *p* value did not reach statistical significance after Bonferroni correction. The other effect related to prior gun experience. When measured using reaction times, people with prior gun experience did not show a gun embodiment effect. However, there was a hint of an effect when we used accuracy instead. To further explore this issue, we conducted Experiment 2, which used the same gun embodiment task but with a different gun.

## Experiment 2

**Fig. 13:**
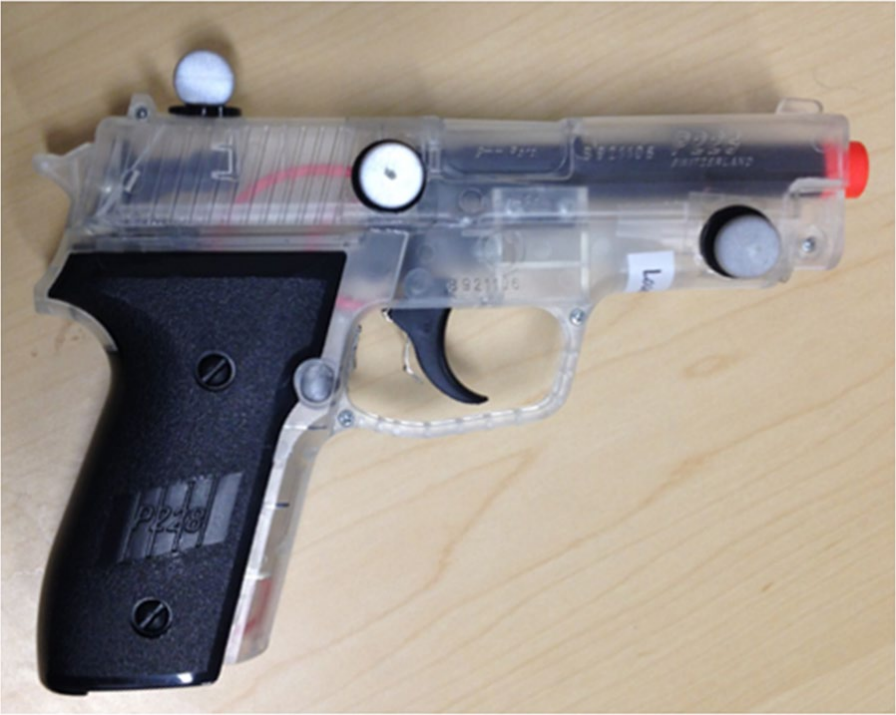
Image of the gun used in the Experiment 2

Often, embodiment effects are stronger, not weaker, for people who have motor expertise (Beilock et al. 2008; Calvo-Merino et al. 2005). In contrast to these reported patterns, the results in Experiment 1 showed a reduction in the gun-embodiment effect for those who had prior experience using a gun, at least when measured using reaction time. This raised the possibility that the plastic toy gun was treated as being like a gun for novices, and thereby producing a gun-embodiment effect, but treated like a toy for those with experience, and thereby not producing a gun-embodiment effect. One way to test this possibility is to use a different kind of gun for the task. In Experiment 2, we administered the same gun perception task as Experiment 1, but instead of a white plastic Wii gun, we used an airsoft gun, which has slightly more resemblance to a real gun (see Fig. 13).

In interest of full disclosure, Experiment 2 was conducted prior to Experiment 1 and therefore was conducted without having a good estimate of the effect size with which to do a power analysis. We recruited many participants who had at least some prior gun experience (*n* = 124) but not many without any gun experience (*n* = 45). We choose to include the data anyway because the results impact how the outcomes from Experiment 1 are interpreted. Specifically, a take-away message from Experiment 1 could be that gun experience is sufficient to prevent a gun-embodiment effect. The results from Experiment 2 invalidate this conclusion.

### Method

The participants were 169 student volunteers from Colorado State University who were recruited in 2014–2015. The images were the same as in Experiment 1. The objects wielded by the participant were an airsoft gun (see Fig. 13) or the spatula used in Experiment 1. The gun perception task was the same as in Experiment 1. Afterward, participants completed a questionnaire that asked whether they owned or had ever used a gun.

### Results

RTs were trimmed by excluding RTs beyond 1.5 times the interquartile range for each participant for each stimulus condition (gun, shoe). Mean RTs were then calculated for each participant for each stimulus condition and for each hold condition (gun, spatula). The gun embodiment score was calculated as the difference in RTs when seeing a gun versus a shoe when holding the gun minus the difference in RTs when holding the spatula. Outliers were determined by having a gun embodiment score beyond 1.5 times the interquartile range for that specific experience group; 9 participants were identified as outliers and removed. Mean RTs are shown in Table 1.

**Table 1:** Mean RTs (and between-subject standard deviations) in ms for Experiment 2

Prior gun experience		Hold gun	Hold spatula
At least once	See gun	574 (78)	585 (79)
	See shoe	614 (77)	615 (74)
None	See gun	567 (74)	570 (68)
	See shoe	607 (78)	601 (82)

There was an insufficient number of participants with no gun experience to have adequate power; only 55% power to detect an effect size of *d*_*z*_ = 0.26 with a one-sided one-sample *t*-test. Thus, we report descriptive statistics only. The mean gun embodiment effect for those with no gun experience was similar to that found in Experiment 1, *M* = 9.30 ms, 95% CI [1.83, 16.67], SEM = 3.70.

**Fig. 14:**
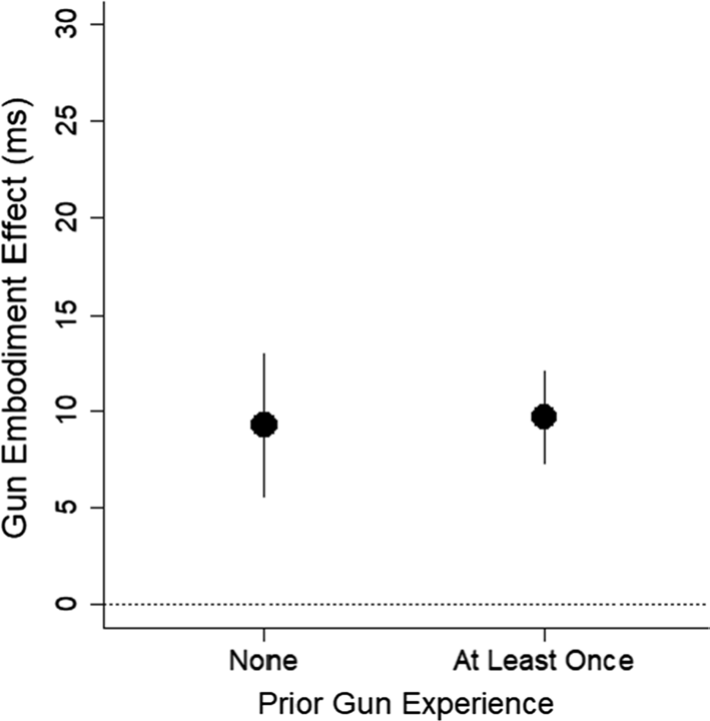
Mean gun embodiment scores as a function of gun ownership and gun gaming status. The dashed line at 0 represents no gun embodiment scores, and more positive values indicate greater gun embodiment scores. Error bars are 1 SEM

The critical test was whether the gun embodiment effect would be observed in people who had prior gun use. We ran a one-sample *t*-test with gun embodiment scores as the dependent measure, which was significant, *t* = 4.06, *df* = 115, *p* < 0.001, *M* = 9.73 ms, 95% CI [4.99, 14.48], BF = 194, *d*_*z*_ = 0.38, 95% CI [0.19, 0.56] (see Fig. 14). This finding is critical because it shows that even people who have prior gun experience can be prone to the gun embodiment effect.

Accuracy (calculated as the proportion of correct responses) is shown in Table 2. Seven participants with at least one prior gun experience were identified as outliers for having difference scores greater than 1.5 times the group’s IQR and were excluded; one participant with no prior gun experience was similarly excluded. Their data are not included in Table 2 or the analyses. For participants with prior gun experience, the gun embodiment effect as measured with proportion of correct responses was approximately 1%, *t* = 2.05, *df* = 108, *p* = 0.043, *M* = 0.012, 95% CI [0, 0.02], BF = 0.79, *d*_*z*_ = 0.20, 95% CI [0.01, 0.39]. For participants with no prior gun experience, the magnitude was similar, *M* = 0.009, 95% CI [− 0.01, 0.03], SEM = 0.01, *n* = 43.

**Table 2:** Mean proportion of correct responses (and between-subject standard deviations) for Experiment 2

Prior gun experience		Hold gun	Hold spatula
At least once	See gun	.94 (.12)	.94 (.13)
	See shoe	.86 (.14)	.88 (.13)
None	See gun	.97 (.03)	.93 (.12)
	See shoe	.88 (.10)	.84 (.17)

Regarding nonparametric signal detection measures, *A*′ scores for each held object for each gun experience group were greater than 0.95, and differences between held object conditions were less than 0.01. This shows that discriminability was high. *B*″, which is a measure of bias, showed a consistent bias to respond that the gun was present (see Table 3). This bias was stronger when holding a gun than when holding the spatula, as shown by the positive difference scores. For the group with prior gun experience, this difference was significant but the evidence for the effect was weak, *t* = 2.25, *df* = 114, *p* = 0.027, *M* = 0.10, 95% CI [0.01, 0.19], BF = 1.15, *d*_*z*_ = 0.21, 95% CI [0.02, 0.39]. There were not enough participants with no prior gun experience to run inferential statistics.

**Table 3:** *B*″ scores (and between-subjects standard deviations) for each held object for both prior experience groups (top two rows) and the difference between *B*″ scores between the held objects for each prior experience group (bottom row)

	Prior gun experience	
	At least once	None
Gun	− .56 (.36)	− .63 (.33)
Spatula	− .45 (.46)	− .46 (.43)
Difference	.10 (.49)	.16 (.36)

More negative *B*″ scores (top two rows) indicate a bias to respond that a gun is present, and more positive difference scores (bottom row) indicate a stronger bias to respond that a gun is present when holding a gun compared with when holding a spatula

### Discussion

The results from Experiment 2 show that people with prior gun experience can be prone to the gun embodiment effect. From a practical perspective, the results from Experiment 2 offer an important advancement, namely that even people with prior experience using guns can be susceptible to the gun embodiment bias. From a theoretical perspective, the primary finding of modulation of the gun embodiment effect found in Experiment 1 was not found in Experiment 2. Instead, there may be some modulation as to whether the gun embodiment effect is expressed in terms of reaction time versus accuracy. Future research is needed to determine when the effect expresses itself in each way.

## General discussion

The gun embodiment effect is the decrement in performance at detecting whether another person is holding a gun or a neutral object when the perceiver also wields a gun. When wielding a gun, perceivers were worse at detecting the presence of a neutral object. The current paper had two goals. The first was to attempt to replicate the original work using a bigger sample size to get a better estimate of the size of the effect. The effect was replicated, albeit in accuracy and speed rather than a specific measure of bias. Across the whole sample, the effect size was, fortunately, small (approximately *d*_z_ = 0.26).

### Gun embodiment effect: replication, magnitude, and measures

Previously, the gun embodiment effect was found with a measure of bias: participants were more biased to report that a gun was present when also holding a gun (Witt and Brockmole 2012). The previous experiments were under-powered, and a *p*-curve analysis raises concern about the veracity of the gun embodiment effect. Given the replication crisis in psychology in general (Open Science Collaboration 2015), concerns about replicability for embodied social cognition in particular (Wagenmakers et al. 2016), and the real-life implications of biases such as the gun embodiment effect, we deemed it necessary to attempt to replicate the gun embodiment effect. In addition to replication, we attempted to pinpoint the magnitude (if any) of the bias.

In the original gun embodiment research (Witt and Brockmole 2012), the gun embodiment effect was found in the nonparametric signal detection measure of bias. Bias measures a tendency in people’s responses or a bias in their perceptions. When holding the gun compared to holding a neutral object, participants were more biased to respond that a gun was present. In the current experiment, there were no differences in bias across the two hold conditions. This reveals a failure to replicate the gun embodiment effect (Witt and Brockmole 2012). However, performance in this task can also be measured in terms of accuracy or in terms of reaction time. With both these measures, the gun embodiment effect was present: when holding a gun compared with holding a spatula, participants were slower and less accurate to respond that a shoe was present. In other words, performance at detecting a neutral object suffered when holding a gun compared with holding a spatula.

The different patterns of results between the original research and the current research raises the question of the true nature of the gun embodiment effect. One possibility is that the nature of the bias has changed over time. The media is currently rife with examples of police officers reporting that they thought they saw a gun being held by what turned out to be an unarmed victim. Perhaps this media exposure has led to a shift in the gun embodiment effect from an influence on bias to an influence on reaction times. However, without specific measures to test this hypothesis and thus no evidence to support it, we find it more likely that the original research mischaracterized the effect in the first place. The original work used small samples sizes, which means the reported results could have reflected spurious effects in bias and missed the small effects on reaction time. The larger sample sizes in the current research encourages greater belief in the present results even though the original work was published first.

The differences between the current findings and the original research raise concerns about the other findings previously published. For example, we had found a similar bias to report that a shoe was present when holding a shoe as the bias to report that a gun was present when holding a gun. This finding challenged the idea that it was the action capabilities of the observer, per se. Instead, the finding suggested the effects may be due to co-representation between objects and actions, as suggested by the common coding approach and the theory of event coding (Hommel et al. 2001; Prinz 1997). We recommend this experiment be replicated to determine whether the effect is specific to being able to act with an object.

The current experiment was powered to be able to find an effect as small as *d*_*z*_ = 0.22. When the effect was measured using reaction times, the effect size was approximately *d*_*z*_ = 0.26, and the difference was approximately 6 ms. With accuracy (measured as the proportion of correct responses), the effect size was approximately *d*_z_ = 0.26, and the difference was approximately 1%. Both measures show effects that are considered small (Cohen 1988). It is possible that decreasing time to respond would increase the impact of wielding a gun on accuracy, as has been shown in other shoot-no shoot paradigms (Correll et al. 2002).

Small effects can have important real-world implications, particularly if they are additive over time (Funder and Ozer 2019). If a policeperson interacts with 10 unarmed people a day and works 250 days per year, given a rate of misjudging a gun to be present at 1%, this would be 25 people who are mistakenly judged to be carrying a gun per policeperson per year. Even if the gun embodiment is characterized as a small effect, it has real-world implications.

### Individual differences and the gun embodiment effect

One of the goals of the present research was to determine whether the gun embodiment effect is universal and fixed or flexible and malleable. Given the real-world implications of the gun embodiment effect, it is important to know if the effect is flexible because this would open the doors to potential developing of training programs. Our strategy to address this question was to assess whether the magnitude of the gun embodiment effect varied as a function of individual differences related to prior gun experience, attitudes, and personality. If the magnitude of the gun embodiment effect was invariant across the individual difference measures, that would provide evidence consistent with a bias that is universal and fixed. In contrast, if the magnitude of the gun embodiment effect varied with even one individual difference measure, this would be evidence for a bias that was not universal and therefore potentially flexible and malleable.

What we initially took as strong evidence for malleability was the significant difference in the gun embodiment effect across people who did and did not have prior gun experience. However, subsequent analyses cast doubt on this difference. In Experiment 1, participants with prior gun experience showed a tendency for a gun embodiment effect in their accuracy scores, and in Experiment 2, participants with prior gun experience showed the gun embodiment effect in reaction times to a similar extent as participants who had no prior experience. We thus have substantial evidence that people with prior gun experience show the gun embodiment effect and cannot argue that prior gun experience modulates the gun embodiment effect.

Across all the other tests for modulation, we found only one measure that possibly correlated with the gun embodiment effect. People who were higher in extroversion than others were more influenced by the gun embodiment effect. However, the effect did reach the criterion for statistical significance after Bonferroni correction.

Despite all the measures, there was little evidence for modulation of the gun embodiment effect. One possibility is that modulation of the effect exists but was too small to detect. Our studies were only powered to 80% to find correlations of *r* = 0.23 or larger. Another possibility is that we did not select the appropriate measures to find modulation of the gun embodiment effect. Given the breadth of our measures, including experience, attitudes, personality, emotion regulation, and impulsivity, we believe the initial exploration included many feasible possibilities, yet few significant correlations emerged. The current evidence is more suggestive of a universal gun embodiment effect than one that is flexible. However, because this was the first attempt to uncover modulation, more research may be needed.

## Summary

The results show that wielding a gun can impact reaction times and judgments of others as also holding a gun. The gun embodiment effect was found both in speed to respond and accuracy of the responses. The preliminary evidence on individual differences is consistent with the idea that the gun embodiment effect is robust and not modulated by personality, prior experience, and a host of other measures. Given the practical importance of finding modulators for use as recruitment criterion or guidance for developing interventions, future research should continue to explore for factors that impact the gun embodiment effect.

## Supplementary information

**Additional file 1**. Multiverse analysis.

## Data Availability

Materials, data, and analysis scripts can be found at https://osf.io/gv2yk/. During peer-review, a read-only link is available at https://osf.io/gv2yk/?view_only=6148493b30694dfd81dd29e0615c38c1.
