# Supplementary material for: Wielding a gun increases judgments of others as holding guns: a randomized controlled trial
Source: Cogn Res Princ Implic. 2020 Nov 16;5:58. doi: 10.1186/s41235-020-00260-3 (PMC7669937; doi:10.1186/s41235-020-00260-3)
Supplement: Supplementary file 1 — Additional file 1. Multiverse analysis. [file 41235_2020_260_MOESM1_ESM.pdf]

Supplementary Materials

The supplementary materials contain the outcomes from the multiverse analysis.

Multiverse Analysis Plan

To eliminate effects of experimenter degrees of freedom, we report a multiverse analysis (Steegen, Tuerlinckx, Gelman, & Vanpaemel, 2016). For a multiverse analysis, the data are presented for each analysis that would have been conducted. For the analysis on the gun embodiment effect, we report a multiverse analysis for which we systematically varied the determination of outliers. We determined outliers at three points in the analysis: raw reaction times (RTs), the bias or difference scores for each hold condition, and the gun embodiment effect (which is the difference in bias or the difference in difference scores between the two hold conditions). For the raw RTs, we used four different criteria. For the *none* criterion, we did not exclude any RTs. For the *fixed* criterion, we excluded RTs faster than 100ms and slower than 1200ms. This criterion matches that reported in Witt and Brockmole (2012), although in retrospect, neither the fixed nor the none criteria are sensible. Thus, the primary focus will be on outlier criteria that are specific to each participant and each stimulus condition. For the *1.5xIQR* criterion, we excluded RTs that were beyond 1.5 times the interquartile range (IQR) for each participant for each stimulus condition. The 3xIQR was the same except we excluded RTs beyond 3 times the IQR. For the difference scores and the gun embodiment effect, we used three criteria: none, 1.5xIQR, and 3xIQR. Altogether, there were 36 different combinations of outlier criteria (4 x 3 x 3). This means that we conducted our critical analysis (e.g., a paired-samples t-test comparing the hold gun condition to the hold spatula condition) 36 times under each of the different outlier criteria.

For the gun embodiment, we also analyzed various dependent measures including a measure of bias, RT, and a measure that combines proportion correct and RT. The outcomes of all are reported as part of the multiverse. For the analysis on individual differences and the gun embodiment effect, we systematically varied the criteria for determining outliers using the 3 criteria of none, 1.5xIQR, and 3xIQR. To the extent that the effect is similar across the multiverse, this is evidence that the effect is robust to outlier exclusion.

Table 1
Multiverse Analysis for B" Scores.

Fixed	1.5xIQR	3xIQR	None	Fixed	1.5xIQR	3xIQR	None	Gun	Diff
**p-value**				**d_z**					
0.73	0.24	0.43	0.82	0.03	0.09	0.06	0.02	3xIQR	**3xIQR**
0.73	0.24	0.43	0.82	0.03	0.09	0.06	0.02	None	
0.61	0.40	0.42	0.75	0.04	0.07	0.06	0.02	1.5xIQR	
0.73	0.24	0.43	0.82	0.03	0.09	0.06	0.02	3xIQR	**None**
0.73	0.24	0.43	0.82	0.03	0.09	0.06	0.02	None	
0.61	0.40	0.42	0.75	0.04	0.07	0.06	0.02	1.5xIQR	
0.54	0.18	0.29	0.69	0.05	0.11	0.08	0.03	3xIQR	**1.5xIQR**
0.54	0.18	0.29	0.69	0.05	0.11	0.08	0.03	None	
0.64	0.36	0.35	0.67	0.04	0.07	0.07	0.03	1.5xIQR	
**BF**				**Raw Units**					
0.09	0.16	0.11	0.09	0.01	0.05	0.03	0.01	3xIQR	**3xIQR**
0.09	0.16	0.11	0.09	0.01	0.05	0.03	0.01	None	
0.1	0.12	0.12	0.09	0.02	0.03	0.03	0.01	1.5xIQR	
0.09	0.16	0.11	0.09	0.01	0.05	0.03	0.01	3xIQR	**None**
0.09	0.16	0.11	0.09	0.01	0.05	0.03	0.01	None	
0.10	0.12	0.12	0.09	0.02	0.03	0.03	0.01	1.5xIQR	
0.10	0.21	0.15	0.09	0.02	0.05	0.04	0.02	3xIQR	**1.5xIQR**

0.10	0.21	0.15	0.09	0.02	0.05	0.04	0.02	None
0.10	0.13	0.13	0.10	0.02	0.03	0.03	0.01	1.5xIQR

Note: The statistical measure for each grid is shown in respective boxes in the top-left corners. Columns show the four outlier criteria for the raw RTs, and rows show the three outlier criteria for the difference scores (Diff) and the gun embodiment effect (Gun). In 3 of the grids, darker colors signify p < .05, $d_z > .20$, or Bayes factor > 3.

Table 2
Multiverse Analysis for RT Scores

Fixed	1.5xIQR	3xIQR	None	Fixed	1.5xIQR	3xIQR	None	Gun	Diff
**p-value**				**d_z**					
0.00	0.00	0.00	0.00	0.21	0.24	0.22	0.22	3xIQR	
0.00	0.00	0.00	0.00	0.21	0.24	0.22	0.22	None	**3xIQR**
0.01	0.00	0.00	0.00	0.21	0.24	0.26	0.24	1.5xIQR	
0.01	0.00	0.00	0.00	0.20	0.24	0.21	0.22	3xIQR	
0.01	0.00	0.00	0.00	0.20	0.24	0.21	0.22	None	**None**
0.01	0.00	0.00	0.00	0.20	0.24	0.24	0.24	1.5xIQR	
0.00	0.00	0.00	0.00	0.23	0.27	0.26	0.26	3xIQR	
0.00	0.00	0.00	0.00	0.23	0.27	0.26	0.26	None	**1.5xIQR**
0.00	0.00	0.00	0.00	0.23	0.27	0.27	0.26	1.5xIQR	
**BF**				**Raw Units**					
4.56	11.92	6.07	5.17	6.80	5.86	6.60	7.94	3xIQR	
4.56	11.92	6.07	5.17	6.80	5.86	6.60	7.94	None	**3xIQR**
2.87	11.92	20.40	11.53	5.79	5.86	6.28	8.01	1.5xIQR	
2.39	12.72	4.07	5.36	6.26	5.94	6.29	7.93	3xIQR	
2.39	12.72	4.07	5.36	6.26	5.94	6.29	7.93	None	**None**
2.60	12.72	11.12	12.03	5.69	5.94	5.96	8.00	1.5xIQR	
8.05	33.87	25.90	28.27	7.36	6.51	7.49	9.30	3xIQR	
8.05	33.87	25.90	28.27	7.36	6.51	7.49	9.30	None	**1.5xIQR**
5.35	33.87	28.03	24.57	6.34	6.51	6.53	8.66	1.5xIQR	

Note: The statistical measure for each grid is shown in respective boxes in the top-left corners. Columns show the four outlier criteria for the raw RTs, and rows show the three outlier criteria for the difference scores (Diff) and the gun embodiment effect (Gun). In 3 of the grids, darker colors signify $p < .05$, $d_z > .20$, or Bayes factor > 3.

Reliabilities

To do individual differences, it is critical to ensure the measure has good reliability. Reliability was calculated by computing a gun embodiment score for the odd trials and the even trials and calculating the correlations between them (see Table 3).

Table 3
Multiverse Analysis for Split-Half Correlations for Reaction Time Scores.

**RTS**					
Fixed	1.5xIQR	3xIQR	None	**Gun**	**Diff**
0.80	0.96	0.86	0.76	3xIQR	**3xIQR**
0.80	0.96	0.86	0.76	None	
0.75	0.96	0.82	0.69	1.5xIQR	
0.80	0.96	0.86	0.76	3xIQR	**None**
0.80	0.96	0.86	0.76	None	
0.75	0.96	0.81	0.69	1.5xIQR	
0.81	0.97	0.86	0.75	3xIQR	**1.5xIQR**
0.81	0.97	0.86	0.75	None	
0.75	0.97	0.82	0.69	1.5xIQR	

Note: Columns show the four outlier criteria for the raw RTs, and rows show the three outlier criteria for the difference scores (Diff) and the gun embodiment effect (Gun). The shading of each cell corresponds to the magnitude of the correlation.

Individual Differences

We conducted a multiverse analysis for the correlation between the gun embodiment effect, as measured with RTs, and each of the individual differences variables. Each analysis was conducted with all participants (*none*), and by removing participants with scores beyond 1.5 times or 3 times the IQR (1.5xIQR, 3xIQR, respectively) on either of the two measures used within each correlation. For example, if someone had a score identified as an outlier on the Big 5 agreeableness measure but not the Big 5 neuroticism measure, their data would be excluded when comparing the gun embodiment effect with agreeableness but not when comparing with neuroticism. The correlations, p-values, and Bayes factors are shown in Table 4.

Table 4. Multiverse Analysis for Correlations between the Gun Embodiment Effect and Individual Difference Scores

Pearson r value			p value			Bayes Factor			Variable
1.5xIQR	3xIQR	None	1.5xIQR	3xIQR	None	1.5xIQR	3xIQR	None	
0.13	0.13	0.13	0.11	0.11	0.11	0.97	0.97	0.97	GAS
0.11	0.11	0.11	0.17	0.17	0.17	0.45	0.45	0.45	Sex
-0.24	-0.24	-0.24	0.00	0.00	0.00	18.38	18.38	18.38	Experience
-0.11	-0.11	-0.11	0.16	0.16	0.16	0.48	0.48	0.48	Stop Signal
0.04	-0.04	-0.06	0.65	0.61	0.50	0.20	0.21	0.23	ISI50
									**Big 5 Scales**
-0.10	-0.12	-0.10	0.19	0.14	0.19	0.42	0.54	0.42	Agree
0.01	0.01	0.01	0.93	0.93	0.93	0.18	0.18	0.18	Open
0.03	0.03	0.03	0.75	0.75	0.75	0.19	0.19	0.19	Conscientious
0.16	0.17	0.16	0.04	0.03	0.04	1.36	1.97	1.36	Extraversion
-0.11	-0.11	-0.11	0.16	0.16	0.16	0.48	0.48	0.48	Neuroticism
									**ED Scales**
-0.01	-0.01	-0.01	0.85	0.85	0.85	0.19	0.19	0.19	Awareness
-0.02	-0.07	-0.03	0.81	0.39	0.74	0.19	0.26	0.19	Impulse
-0.12	-0.12	-0.12	0.13	0.13	0.13	0.54	0.54	0.54	Nonaccept
-0.01	-0.05	-0.01	0.85	0.54	0.85	0.19	0.22	0.19	Clarity
-0.02	-0.02	-0.02	0.84	0.84	0.84	0.19	0.19	0.19	Goals
-0.11	-0.11	-0.11	0.17	0.17	0.17	0.45	0.45	0.45	Strategies
									**SSPT**
-0.07	-0.10	-0.10	0.32	0.19	0.19	0.29	0.41	0.41	ES
-0.06	-0.06	-0.06	0.46	0.46	0.46	0.24	0.24	0.24	RS
									**LOC Scales**
-0.02	-0.04	-0.02	0.81	0.62	0.81	0.18	0.20	0.18	Chance
-0.07	-0.06	-0.07	0.37	0.41	0.37	0.25	0.24	0.25	Internal
-0.05	-0.05	-0.05	0.54	0.54	0.54	0.21	0.21	0.21	Power Other
									**UPPS Scales**
0.03	0.06	0.03	0.68	0.47	0.68	0.20	0.24	0.20	Premeditation
-0.07	-0.07	-0.07	0.36	0.36	0.36	0.27	0.27	0.27	Perseverance
-0.02	-0.03	-0.02	0.83	0.70	0.83	0.19	0.20	0.19	Neg Urgency
0.07	0.07	0.07	0.39	0.39	0.39	0.26	0.26	0.26	Pos Urgency

Note: Values in the columns correspond with Pearson *r* value, corresponding *p* value, and the Bayes Factor when the gun embodiment effect (measured as reaction time) is correlated with each

individual difference across 3 outlier criteria. Shading darkness corresponds to absolute *r* values greater than .20, *p* values less than .05, and Bayes factors greater than 3.

Given that people who have used a gun at least once did not show the effect, it seemed reasonable to re-evaluate the correlations with only participants who reported having never used a gun. The corresponding multiverse is in Table 5.

Table 5. Multiverse Analysis for Correlations between the Gun Embodiment Effect and Individual Difference Scores

Pearson r value			p value			Bayes Factor			Variable
1.5xIQR	3xIQR	None	1.5xIQR	3xIQR	None	1.5xIQR	3xIQR	None	
-0.04	-0.04	-0.04	0.71	0.71	0.71	0.25	0.25	0.25	GAS
-0.01	-0.01	-0.01	0.33	0.33	0.33	0.36	0.36	0.36	Stop Signal
0.04	-0.05	-0.09	0.71	0.66	0.37	0.25	0.26	0.35	ISI50
									**Big 5 Scales**
0.04	0.04	0.04	0.72	0.752	0.72	0.25	0.25	0.25	Agree
0.03	0.03	0.03	0.77	0.77	0.77	0.24	0.24	0.24	Open
0.15	0.15	0.15	0.15	0.15	0.15	0.62	0.62	0.62	Conscientious
0.27	0.31	0.27	0.01	0.00	0.01	7.84	20.95	7.84	Extraversion
-0.28	-0.28	-0.28	0.01	0.01	0.01	8.84	8.84	8.84	Neuroticism
									**ED Scales**
-0.09	-0.09	-0.09	0.37	0.37	0.37	0.34	0.34	0.34	Awareness
-0.11	-0.17	-0.11	0.28	0.10	0.28	0.40	0.88	0.40	Impulse
-0.12	-0.12	-0.12	0.24	0.24	0.24	0.44	0.44	0.44	Nonaccept
-0.13	-0.16	-0.13	0.19	0.12	0.19	0.53	0.72	0.53	Clarity
-0.11	-0.11	-0.11	0.26	0.26	0.26	0.42	0.42	0.42	Goals
-0.19	-0.19	-0.19	0.06	0.06	0.06	1.16	1.16	1.16	Strategies
									**SSPT Scales**
0.01	0.01	0.01	0.96	0.96	0.96	0.23	0.23	0.23	ES
-0.02	-0.02	-0.02	0.84	0.84	0.84	0.24	0.24	0.24	RS
									**LOC Scales**
-0.07	-0.10	-0.07	0.47	0.33	0.47	0.30	0.36	0.30	Chance
-0.06	-0.06	-0.06	0.52	0.53	0.52	0.28	0.28	0.28	Internal
-0.08	-0.11	-0.08	0.45	0.26	0.45	0.30	0.42	0.30	Power Other
									**UPPS Scales**
0.03	0.06	0.03	0.76	0.57	0.76	0.24	0.27	0.24	Premeditation
-0.14	-0.14	-0.14	0.16	0.16	0.16	0.60	0.60	0.60	Perseverance
-0.05	-0.07	-0.05	0.62	0.51	0.62	0.26	0.28	0.26	Neg Urgency
0.04	0.04	0.04	0.73	0.73	0.73	0.24	0.24	0.24	Pos Urgency

Note: Values in the columns correspond with Pearson *r* value, corresponding *p* value, and the Bayes Factor when the gun embodiment effect (measured as reaction time) is correlated with each

individual difference across 3 outlier criteria. Shading darkness corresponds to *r* values greater than .20, *p* values less than .05, and Bayes factors greater than 3.

References

Steegen, S., Tuerlinckx, F., Gelman, A., & Vanpaemel, W. (2016). Increasing Transparency Through a Multiverse Analysis. *Perspectives on Psychological Science*, *11*(5), 702–712. https://doi.org/10.1177/1745691616658637
